# Selections and Abstracts

**Published:** 1898-01

**Authors:** 


### *SELECTIONS AND ABSTRACTS.*

---

### How to Cook a 'Possum.

A lady, lately returned from New Orleans, tells this story (*Philadelphia Times*):

She was walking down Chartres street early one morning, intending to visit the celebrated French market of the Crescent City, and on her way she met a very old colored man coming from the opposite direction, evidently from the market, as he was carrying in one hand a 'possum and in the other a small split-wooded basket of sweet potatoes. The old man's face was beaming with good nature and wreathed in smiles of anticipatory pleasure. He looked so joyously into the face of the lady that she, too, could not help but smile at him, whereupon he held the 'possum up aloft and said, "Good eatin', missey, good eatin'." She stopped for a moment, looked at the childlike, happy face of the old negro, and said:

"So you like 'possum, do you?"

"Like 'possum, missey! I loves 'possum. Dare ain't no eatin' like 'possum. De 'possum am good, but de gravy with sweet potatoes is better. Did you never eat 'possum, missey? Den you didn't know what good eatin' was. But mebbe you all wouldn't know how to cook 'possum, fur dars ebbyt'ing in de know how."

"Well, then, tell me how you cook it," she said.

The old man sat the 'possum and potatoes down on the pavement, and with a look of earnest concentration, began: "Now don't you never forget jest what I'se gwine to tell you about how to cook de 'possum. Well, de fust t'ing you does is to get you 'possum. Dat may be easy for you'ins, but tain't for me; dat is, always. Well, den, when you's dun got you's 'possum you cleans him fust. Den you puts him into de pot with cold water, and put de pot over a hot fire an' den you parbiles him—not too much—fur you don't want to loose any of his nice sweet fat. Den you takes him out of de pot an' you dries him in a clean towel. Den you puts him in a big frying-pan; den you scrapes de skin off you sweet potatoes an' you puts dem into de same pan wid de 'possum. Den you has you stove red, and den you puts de pan an' 'possum an' potatoes into de oven and den go away for a little while, but not too long. Den when you come back you puts in a little hot water, an' den you begins and bastes de 'possum an' de sweet potatoes an' you keeps on a-basting and a-basting till de 'possum is a good brown—jest like my color—an' de sweet potatoes is soft and juicy an' de gravy is almost black, and plenty of it. Den you takes it out ob de oven an' den you sots de table, and den—well den, you bars de doors, fo' the smell of cooked 'possum goes a long ways, an' when you have only one 'possum you doesn't want much company besides yourself."

Now, there is your recipe for cooking 'possum, and by probably one of the most skillful chefs for that dish in the world.

---

### SOME LESSONS OF THE YELLOW FEVER EPIDEMIC.

Americans do not sufficiently realize what a humiliating reflection upon the enlightenment of the western hemisphere, from a sanitary point of view, a yellow fever epidemic constitutes. In Europe, the disease is now practically unknown; the last serious epidemic having been that of Lisbon in 1857.

Yellow fever now belongs to the western hemisphere, from which also it should be extirpated, as can be done, if the proper kind of international public opinion be brought to bear upon the subject. While the disease is not indigenous to the United States, being always an importation, its visits are, nevertheless, so regular, that since the beginning of the century there have been only nine years in which it has not appeared here. Its natural breeding ground is furnished by the heat, moisture and filth of tropical sea-ports. These conditions are found in certain cities of the Spanish main, which lacking proper sanitary regulations, have become perpetual foci of infection; and Havana is the worst of them. There the disease prevails during the entire year, a steady supply of fuel for its virulent flames, as it were, being furnished by the newcomers.

The natives generally are immune to it, having usually had the malady, in a mild form in childhood.

For us the position is a simple one: Since we have such neighbors, we must either bring about a reform in their sanitary conditions and practices, or continue to run the risk of an annual invasion of this terrible disease. Thirty-five of the visits of yellow fever to this country since 1800 are known definitely to have been from Cuba; and of these, twenty-three have been clearly traced to the port of Havana. Europe's protection against Cuba in this particular lies in her remoteness. A disease which lurks in a vessel starting across the ocean has time to develop and manifest itself so clearly that the quarantine officials on the other side can discover it on the vessel's arrival. But with Cuba hardly six hours from Key West there will always be a percentage of danger, however stringent the quarantine regulations may be, if the conditions remain as they are, unless indeed we assume a policy of absolute non-intercourse with the island during the summer months.—*Dr Walter Wyman in Forum.*

---

### "TYPHO-MALARIAL FEVER" BECOMING OBSOLETE.

For many years the question as to the existence or non-existence of an alleged hybrid fever, that was claimed to be neither typhoid nor malarial, has been discussed, *pro* and *con*, without definite results either way. While the best minds in the profession have contended against a specific third form of fever, their assertions lacked essential proof, and the question remained unsettled.

In the *North Carolina Medical Journal* of August 5, 1897, Dr. H. A. Royster, of Raleigh, contributes an article on the "Practical Results in the Diagnosis of Continued Fevers from Examination of the Blood" that sheds convincing light on this subject. During the past year Dr. Royster has had under observation thirty cases of continued fever, all of which he subjected to an examination for the plasmodium malariæ. In consequence of this examination he was enabled to cure with quinine every case in which the plasmodium was discovered, and, on the other hand, every case in which the result of the examination was negative developed into clinically true typhoid fever. In view of his observations, extending over a considerable period of time, Dr. Royster feels justified in asserting that the continued fevers of the South are either typhoid or malarial.

We believe that the term typho-malarial will soon be relegated to the obsolete list, for with the cause of both typhoid and malarial fevers known and capable of demonstration, the ante-bacterial and protozoal theories held by clinicians must necessarily fall when unsupported by modern microscopical investigation.—*Pa. Med. Journal.*

---

### FOOTBALL.

In view of the great number of accidents on the football field between college teams, it is impossible any longer to view the game in the light of innocent recreative amusement with harmless and healthful athletics as its object. Although so-called slugging has been ruled out in the new game, there is still left enough of brutal muscular force to make the alleged sport productive of the greatest variety of surgical injuries to every part of the body. In fact there is hardly a game played in which some one of the contestants is not more or less seriously hurt. Only the severer injuries are noted, while the lesser ones serve as enlivening incidents to call forth the plaudits of an excited audience. Short of actual death on the field, not much account is taken of the hundreds of young men who are oftentimes injured for life as the result of the rough-and-tumble methods of the match. The trainers explain the number of injuries by the lack of requisite physical preparation for the contest, but in reality the more the footballers are trained the more dangerous becomes the game. It is certainly time we should look the matter fairly in the face. If we wish to develop pluck, courage, endurance, and strength, we can do so in more healthful and safer ways. It is time that the new game, with mere weight against weight, should be abolished.—*Medical Record.*

---

### SUBSTITUTE FOR ALCOHOL IN ALCOHOLIC NEURASTHENIA.

℞ Tr. nuc vomic........................................ ...................ʒi. + mxx.

Spts. ammon. aromat. Hoffman's anodyne........ ..... aa. ℥ij.

Sig. ʒij. pro. re nata—*M. A. Starr.*

### FORMALDEHYDE GAS.

Dr. H. C. Wood states that the chief interest in this drug centers in its powers as a germicide and disinfectant. As a gas it will penetrate not only animal tissues, but almost all organic substances, so that books infected with various pathogenetic germs could be disinfected by being shut up for fifteen minutes in an atmosphere containing the vapor of commercial formalin (40 per cent. aqueous solution of formaldehyde), one part to 300 of air. The method of Trillat is the preferable one, and consists in the use of the formaldehyde directly after its production by the passage of the vapors of methylic alcohol over red-hot metal. Kinyoun has shown that none of the ordinary fabrics are injured by the gas, which is capable of completely disinfecting curtains, carpets, clothing, bed-covering, and the minor forms of furniture, although it is doubtful whether heavy upholstered furniture, such as sofas and mattresses, can, in their interior, be thoroughly disinfected. The gas is so irritating that no one can remain in the room during the disinfection, but the lamp employed is automatic and can be left to itself. It can also be used for the removal of foul odors; $\frac{1}{2}$ to 1 per cent. solution is sufficient for cleansing vessels in the anatomical library. If the hands be washed with it and afterwards with alcohol, they are rendered completely antiseptic, but are not stained or irritated. It does not affect instruments, and it is efficient in preparing catgut and surgical dressings. For the cleansing of an infected wound a 2 per cent. solution is used, but for a continuous local application or free irrigation, one-fourth of 1 per cent. is sufficient.—*University Medical Magazine.*

---

PELLMAN, of the University of Bonn, tells of a notorious drunkard who died in 1800. Her descendants numbered 834 and 709 of them have been traced. Of these, 7 were convicted of murder, 76 of other crimes, 142 were professional beggars, 64 lived on charity, and 181 women of the family were prostitutes. This single family cost the German government, through its courts, almshouses and prisons, no less a sum than $1,250,000.—*Med. and Surg. Reporter.*

---

### GOOD SALVE FOR HEMORRHOIDS.

| | | |
|---|---|---|
| ℞ | Cocaine muriate | gr. xx. |
| | Morph. sulph. | gr. v. |
| | Atrop. sulph. | gr. iv. |
| | P. tannin | gr. xx. |
| | Vaselini | ℥ i |
| | Ol. rosæ | q. s. |

M. Sig. Apply after every evacuation—*Ex.*

---

### FOR NEURASTHENIA.

| | | |
|---|---|---|
| ℞ | Potass. bromidi | ℥ iss. |
| | Ammon | ʒ iss. |
| | Potass. bicarb. | gr. xiii. |
| | Tr. calumbæ | ℥ iss. |
| | Aquæ | ℥ vi. |

Sig. Teaspoonful in water 3 times daily.—*A. D. Rockwell.*

---

Nurse—"Johnnie, the stork has just brought you a little baby. Wouldn't you like to see your little brother?"

Johnnie—"Naw, but I'd like to see the stork."—*Colorado Med. Journal.*

---

I HAVE given your Bromidia with success as a remedy for insomnia, especially where produced by excessive study or mental work.

DR. LUIGI SALUCCI,

Physician to the Holy Apostolic Palaces, the Vatican, Rome.

